# Efficient spatial and channel net for lane marker detection based on self-attention and row anchor

**DOI:** 10.1038/s41598-023-47071-2

**Published:** 2023-11-20

**Authors:** Shengli Fan, Yuzhi Zhang, Shengrong Lu, Xiaohui Bi

**Affiliations:** 1 Department of Automotive Engineering, Hebei College of Industry and Technology, Hongqi Avenue 626, Shijiazhuang, 050091 China; 2 College of Mathematics and Information Engineering, LongYan University, Dongxiaobei Road 1, Longyan, 364000 China

**Keywords:** Computational science, Computer science

## Abstract

Lane detection is an important component of advanced driving aided system (ADAS). It is a combined component of the planning and control algorithms. Therefore, it has high standards for the detection accuracy and speed. Recently several researchers have worked extensively on this topic. An increasing number of researchers have been interested in self-attention-based lane detection. In difficult situations such as shadows, bright lights, and nights extracting global information is effective. Regardless of channel or spatial attention, it cannot independently extract all global information until a complicated model is used. Furthermore, it affects the run-time. However trading in this contradiction is challenging. In this study, a new lane identification model that combines channel and spatial self-attention was developed. Conv1d and Conv2d were introduced to extract the global information. The model is lightweight and efficient avoiding difficult model calculations and massive matrices, In particular obstacles can be overcome under certain difficult conditions. We used the Tusimple and CULane datasets as verification standards. The accuracy of the Tusimple benchmark was the highest at 95.49%. In the CULane dataset, the proposed model achieved 75.32% in F1, which is the highest result, particularly in difficult scenarios. For the Tusimple and CULane datasets, the proposed model achieved the best performance in terms of accuracy and speed.

## Introduction

Autonomous driving is a complex process that involves a variety of sensors such as cameras, lidar and radar and requires increasingly complex models and algorithms. The aim is to fully understand the environment in order to be able to take appropriate measures. An extremely important part of vehicle control is the lane marking line. Some processes such as lane keeping and highway assistance are highly dependent on it. In addition, it is essential for regional planning and vehicle control. Therefore, increasing importance is being given to improving the time-of-flight response and accuracy of vision-based lane marking detection^1–6^.

The fundamental problem in lane marking identification, as previously mentioned^7–9^, is how to accurately detect the lane line under difficult circumstances. As a result of a lack of visual cues, including significant vehicle occlusion, harsh lighting, shadows and wet conditions, errors or false alarms go unnoticed. Traditional vision-based methods^10^ are mainly based on hand-crafted features, gray images, ROIs and various edge detection operators such as SIFT^11^ and SURF^12^. However, their ability to adapt to difficult weather conditions and harsh lighting conditions is inadequate. This prevents wide generalization and use. CNN has attracted a lot of attention in recent years. It works well in extracting features. However, to achieve high performance in classification and regression, it is necessary to make a trade-off between the receptive field and the network depth. A2-Net^13^, Squeeze and Excitation Networks^14^, CBAM^15^ and Gather-Excite^16^ are examples of attention and self-attention mechanisms^17,18^ that have been developed and advanced using technology support the detection of lane markings. It can spatially focus attention on multiple areas or channel attention to comprehensively extract broad information.

Spatial self-attention focuses on spatial relationships rather than channel co-relations. Instead, channel self-attention emphasizes channel rather than spatial dependence and we thoroughly examine the benefits of channel attention and spatial attention to understand many facets of self-attention. We suggest that ESCN is an effective spatial and channel network. The main contributions of our proposed model are summarized as follows:

1. A brand new ESCN mechanism. To build a novel ESCN model, we merged spatial and channel self-attention based on the anchor representations. It can fully utilize channel and spatial correlations simultaneously to extract global information, especially under difficult conditions.
2. A powerful, lightweight design. To avoid dimensionality reduction, we recommend 1D and 2D convolutions across channels and spaces.
3. Numerous experiments. Our benchmarks are CULane and Tusimple. The results show that our proposed methodology provides state-of-the-art performance through intensive visualization and experiments.

## Related work

First, computer vision was the primary research method. With the advancement of CNN and transformer technologies, lane mark detection technology is receiving increasing attention in science. The three main areas of interest of research and major achievements are as follows:

1. Tradition approaches based on vision. The primary technologies at this level are vision-based methods^19–26^. It includes three sectors: the model-based approach in one, the feature-based method in the other two, and the region-based method in the third. Image segmentation, vanishing point selection, orientation estimation and lane detection are the four processes that typically involve model-based techniques. During the image segmentation step, the entire image was divided into a near field and a far field as separate ROIs. Uses^27^ the Gaussian model^28^ and maximum likelihood to estimate the vanishing point and presents the Gabor filter to estimate the orientation. The Canny edge detector, Hough transform, Catmull-Rom spline, spline model, cubic spline, IPM and particle filter are also used in track-bound detection. The three feature-based techniques include feature extraction, line detection and tracking. To extract features from an ROI^29^, a local thresholding technique was proposed that uses template matching for line detection. The EKF was proposed in^30–33^ for lane marking tracking. The region-based approach includes both region finding and feature tracking. The Shi-Tomasi method was proposed by^33^ for feature extraction, while the Lucas-Kanade tracker and optical flow algorithm were proposed separately by^34^ and^35^, respectively.
2. Segmentation approach using CNN. Several groups are currently working intensively on applying CNN techniques^36,37^ to lane marker detection^38,39^. Similar to R-CNN, CNNs are data-driven and suitable in feature extraction^40^. Although deep learning-based methods such as CNN^41^ include numerous convolution and pooling layers, they cannot fully utilize the information and context, especially in difficult situations such as occlusion, lane marking degradation, and changing road conditions. Semantic segmentation^42^ and instance segmentation^43^ have been proposed as solutions to this problem^44^ proposed pixel-level semantic segmentation to identify lane markings as a step in semantic segmentation. Proposes a UNet-based weakly supervised lane marking detection network^45^. In contrast to semantic segmentation^46^, presents an end-to-end lane mark detection based on instance segmentation, which consists of a lane segment branch and a lane embedding branch to increase the speed of lane mark detection. Proposed a fast structured track identification network that selects regions with given lines instead of the entire image to avoid extensive processing^47^.
3. With CNN + attention. Detecting lane markings in difficult situations is a significant problem. CNN + segmentation techniques were successful but encountered significant challenges. For example, significant computational effort is required to use semantic segmentation-based methods, and the accuracy of lane lines and the number of lane lines need to be promoted and improved. Therefore, attention has been paid to attention-based methods for lane marking detection^48–53^. In^52^, an ESA module based on encoder and decoder architecture was proposed. In order to be able to determine the position of the occlusion more precisely, HESA and VESA were integrated. Proposed to use spatial attention to collect boundary information across multiple locations and channel attention in the GCE module to extract information about the global context^50^. For U-Net^51^, proposed residual blocking and attention mechanisms.

Comparisons between the above methods can be found in Table 1.

**Table 1:** Comparisons among different methods of lane mark line detection.

Methods	Strength	Limitation	Performance
Tradition approaches based on vision	(1) Simple and convenient	(1) Only depend on edges, color, thickness and shape to detect lanes	(1) For simple scenarios
	(2) The computing power of the embedded platform is not relatively high	(2) The adaptability of the algorithm is not strong	(2) Accuracy and F1 indicators are not very high
		(3) Result in a lot of work and low robustness. When the driving environment changes significantly, the effect of lane line detection is not good	(3) Speed of FPS is generally is generally fast
		(4) Be sensitive to changes in light, weather conditions and noise. When the external environment changes significantly, many traditional lane detection systems fail	
Segmentation approach using CNN	(1) Lightweight and efficient networks	(1) Require more computing resources	(1) Higher accuracy and better robustness
	(2) Particularly suitable for embedded systems and real-time applications	(2) The model is large and the processing speed is generally slow	(2) Suitable for urban roads
		(3) Under strong obstacles, performance was poor and prior knowledge of the lane line was not fully utilized	(3) Speed of FPS is not too high
With CNN + attention	(1) Emphasizing local information and global information	(1) Higher computing power requirements	(1) Suitable for complex scenarios
	(2) Easily to handle the obstruction, lack or weak display of the lane lines in complex scenes	(2) Network model is more complicated and big	(2) Higher accuracy and F1 indicators
	(3) Better real -time	(3) Requiring more memory resources	(3) Speed of FPS is high in the case where the computing power is guaranteed

## Proposed approach

### System overview

A2-Net^13^, Squeeze and Excitation Networks^14^, CBAM^15^ and Gather-Excite^16^ are examples of attention and self-attention mechanisms^17,18^ that have been developed and advanced using technology support the detection of lane markings. It can spatially focus attention on multiple areas or channel attention to thoroughly extract global information.

**Figure 1:**
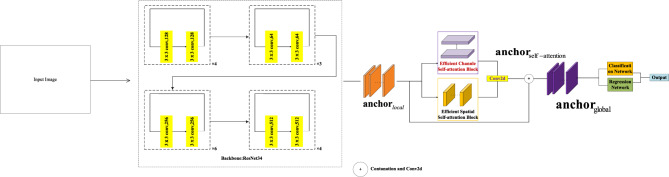
The Architecture of ESCN.

Spatial self-attention focuses on spatial relationships rather than channel connections. Instead, channel self-attention emphasizes channels rather than spatial dependence, and we thoroughly examine the benefits of channel and spatial attention to understand many facets of self-attention. We propose that the ESCN is an effective spatial and channel network, as shown in Fig. 1. The main contributions of our proposed model are summarized as follows:

As mentioned above, lane marking detection is difficult to solve in challenging scenarios such as severe lane erosion, strong shadow and vehicle occlusion. To overcome these problems, we introduce a lightweight and efficient channel attention model that extracts feature maps from DCNN as inputs. To obtain global semantic and contextual information, we use cross-channel to match anchor vectors in its own channel and its neighbors. It can capture cross-channel interactions to learn effective and efficient channel attention while avoiding dimensionality reduction, as shown in Fig. 1. Therefore, it can summarize and abstract all this global information without changing the receptive field. In addition, it promotes classification precision and location accuracy at the same time.

### Backbone

We used ResNet34 as the backbone of our proposed ESCN. There are four different types of residual blocks. The size of their convolution kernel was 33, and their individual kernel and channel numbers were 64, 128, 256, and 512, respectively. This successfully prevented the gradient from disappearing or exploding.

### Efficient channel and spatial attention block

The heart of the ESCN is an efficient channel and spatial attention block. There are two types of attention systems. One is an effective block of channel attention, the other is an effective block of spatial attention. This combination is about placing global contextual information alongside global location information in a single channel. Therefore, lane marking features can be effectively extracted even in difficult situations.

1. Efficient Channel Attention Block. After extracting the feature maps using of ResNet34, the local feature map ${\mathbf{anchor}}_{local} \in {\mathbf{R}}^{C \times H \times W}$ served as the input. $C,H,W$ indicate the channel number, feature-map height and feature-map width respectively. Then global average pooling operates on it as shown in (1):
  1 $$ f({\mathbf{X}}_{k} ) = \frac{1}{H \times W}\sum\limits_{i,j = 1}^{H,W} {x_{k}^{i,j} } \,({\mathbf{X}}_{k} \in {\mathbf{anchor}}_{local} )\; $$
  where $k = 1,2,3, \ldots ,C$ and $y_{k} = f({\mathbf{X}}_{k} )$. So we get ${\mathbf{Y}} = \left( {y_{1} ,y_{2} ,y_{3} , \ldots y_{i} , \ldots ,y_{j} , \ldots ,y_{C} } \right)^{T}$.

**Figure 2:**
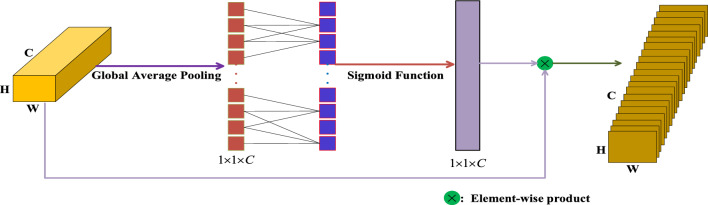
The architecture of efficient channel attention block.

**Figure 3:**
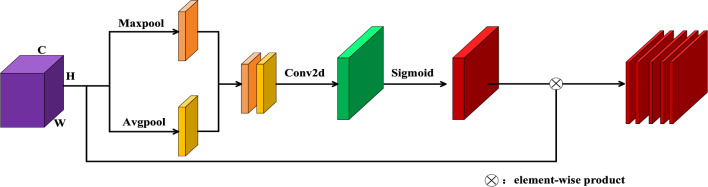
The architecture of efficient spatial attention block.

We also know that many parameters are involved in the linear transformation. Regardless of whether it is a full or diagonal matrix, this results in numerous computations. To avoid this, we propose a 1D convolution with kernel size $k$. It is shown as follows:

2 $$ {\mathbf{W}} = {\text{Re}} lu(C1D_{k} ({\mathbf{Y}})) $$

where Relu indicates the Rectified Linear Unit and $C1D_{k}$ is the 1D convolution which involves $k$ parameters. Therefore it reduces the number of parameters and computation time. It can be easily observed that $k$ represents the local cross-channel interaction. This is a key factor in reducing the parameter quantity. To avoid manual tuning, we adopted the following adaptive expression^54^:

3 $$ k = \psi (C) = \left| {\frac{{\log_{2}^{C} }}{\gamma } + \frac{b}{\gamma }} \right|_{odd} \; $$

where $k$ is an odd number and $C$ indicates the channel number. In our proposed model, we set $\gamma$ and $b$ as 2 and 1 respectively. The detailed architecture is shown in Fig. 3. Finally, we obtain the output of efficient channel attention which indicates ${\mathbf{anchor}}^{{{\text{ch}}annel\_attention}}$. The structure is shown as Fig. 2.

2. Efficient Spatial Attention Block. We know that the local feature map ${\mathbf{anchor}}_{local}$ is put into channel attention and spatial attention blocks. Then in the efficient spatial attention block the local feature map ${\mathbf{anchor}}_{local}$ is applied with Maxpool and AvgPool operations as follows:
  4 $$ anchor_{i,j}^{spacial,\max pool} = \mathop {\max }\limits_{k \in C} ({\mathbf{X}}_{k,i,j} )\;(i \in H,j \in W) $$
  where the $anchor_{i,j}^{spacial,\max pool}$ indicates the value at location $(i,j)$ after the maxpool operation. And we get the feature map ${\mathbf{anchor}}^{spatial,\max \_pool}$. Equation (5) is expressed as follows:
  5 $$ {\mathbf{anchor}}^{spatial,\max \_pool} = \left( {\begin{array}{*{20}c} {anchor_{0,0}^{spatial,\max \_pool} } & {...} & {anchor_{0,W - 1}^{spatial,\max \_pool} } \\ {...} & {...} & {...} \\ {anchor_{H - 1,0}^{spatial,\max \_pool} } & {...} & {anchor_{H - 1,W - 1}^{spatial,\max \_pool} } \\ \end{array} } \right) $$

Avgpool is shown as follows:

6 $$ {\mathbf{anchor}}_{i,j}^{{spacial,{\text{avg}}pool}} = \frac{1}{C}\sum\limits_{k = 1}^{C} {{\mathbf{X}}_{k,i,j} } (i \in H,j \in W) $$

where $anchor_{i,j}^{{spacial,{\text{avg}}pool}}$ are the location value positions $(i,j)$. After the avgpool operation we obtained the feature map ${\mathbf{anchor}}^{spatial,avg\_pool}$.

7 $$ {\mathbf{anchor}}^{{spatial,{\text{a}}vg\_pool}} = \left( {\begin{array}{*{20}c} {anchor_{0,0}^{spatial,avg\_pool} } & {...} & {anchor_{0,W - 1}^{spatial,avg\_pool} } \\ {...} & {...} & {...} \\ {anchor_{H - 1,0}^{spatial,avg\_pool} } & {...} & {anchor_{H - 1,W - 1}^{spatial,avg\_pool} } \\ \end{array} } \right) $$

After we obtain the ${\mathbf{anchor}}^{spatial,\max \_pool}$ and ${\mathbf{anchor}}^{spatial,avg\_pool}$, we concatenated them. Then we apply Conv2d with kernel size 3 and a sigmoid function Finally we acquire the output of the efficient spatial attention block which is the ${\mathbf{anchor}}^{spatial\_attention}$. The detailed architecture is illustrated in Fig. 3.

### Classification network and regression network

**Figure 4:**
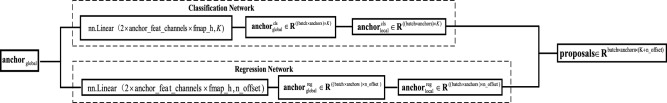
The Architectures of Classification Network and Regression Network.

${\mathbf{anchor}}_{global}$ is input into Classification networks and Regression networks separately. Each network passes through a linear layer and reshaping operation. They then join together to become a tensor ${\mathbf{proposals}}\left( {{\mathbf{proposals}} \in {\mathbf{R}}^{batch \times anchors \times (K + n\_offset)} } \right)$ where $K$ is the classification number and $n\_offset$ represents the offset number in $X$ coordinate frame. Finally the ${\mathbf{proposals}}$ are performed iteratively using a non-maximum suppression NMS (Non Maximum Suppression) operation in batch dimensions. The softmax operation is performed to ${\mathbf{score}}[:,2]({\mathbf{scores}} \in {\mathbf{R}}^{anchors \times (K + n\_offset)}$ which in our study K is set as 2. Therefore in $anchor$ rows we find out whose probabilities are greater than conf_threshold, which is a possible threshold for judging whether it is a lane marking. In this way we obtained the classification results. We also find the anchor position index, which represents the regression result, because each classification and regression component is in the same row and positions different columns. The detailed architecture of the classification and regression networks is shown in Fig. 4. The loss function is given by (8):

8 $$ f\left( {\{ c_{i} ,a_{i} \}_{i = 0}^{{N_{a} - 1}} } \right) = k_{c} \sum\limits_{i} {\Psi_{class} \left( {c_{i} ,c_{i}^{*} } \right)} + k_{r} \sum\limits_{i} {\Phi \left( {a_{i} ,a_{i}^{*} } \right)} \; $$

where $c_{i} ,a_{i}$ are the prediction results of the classification and regression respectively, $c_{i}^{*} ,a_{i}^{*}$ are the ground truths for the anchor $i$. $N_{{\text{a}}}$ is the total number of anchors. $k_{c} ,k_{r}$ are the coefficients of the classification and regression loss functions respectively, and are used to balance the loss value. In the proposed model $k_{c} = 10$ and $k_{r} = 1$. Meanwhile we also set $\Psi_{{{\text{cla}}ss}}$ to Focal Loss^54^ and take $\Phi$ as Smooth L1 individually.

## Experiments

### Dataset

To demonstrate the effectiveness of the model and evaluate the results of our proposed methodology, we used two commonly used benchmark datasets, TuSimple^55^ and CULane^1^. Most highway scenarios of the TuSimple dataset. Due to the uniform illumination, it is much easier to detect the lane marking line, while the CULane dataset is far more complicated than the previous one. Nine difficult scenarios were considered: crowd, no queue, normal, blinding night, shadow, curve and arrow in city and highway environments. Table 2 provides a detailed explanation of the two data sets.

**Table 2:** Overview of dataset description used in this paper.

Dataset	#Frame	Train	Validation	Test	Resolution	#Lane	#Scenarios	Environment
Tusimple	6408	3268	358	2782	1280 × 720	≤ 5	1	Highway
CULane	133,235	88,880	9675	34,680	1640 × 590	≤ 4	9	Urban and highway

### Evaluation metrics

The TuSimple and CULane benchmarks use different evaluation metrics. Accuracy served as the evaluation standard for the TuSimple benchmark.

For the CULane benchmark, the final evaluation metric was the $F1$ combined with two other metrics:${\text{Precision}}$ and ${\text{Recall}}$.

### Implementation details

In the experiment, all images were resized to $360\; \times \;640$ pixels for TuSimple and CULane respectively. Therefore $H$ and $W$ were set as 360 and 640 respectively. The epoch was set to 50 for the TuSimple benchmark and 15 for CULane. The batch size was set to eight and the learning rate was set to 0.0003 using the Adam optimizer. We use pthon3.7, pytorch 1.6.0, cuda 10.1 and Cudnn 7.2 as the experimental environment.

### Results

1. Results on TuSimple dataset.

The accuracy of the proposed model is 95.49% for the TuSimple benchmark and 95.12% for the top technique. The accuracy of our model was improved by 0.37% compared to the other techniques. The proposed model had FP and FN values of 0.0307 and 0.0342, respectively. The former has the lowest values for all methods and there is hardly a gap of 0.0118. Thus, the proposed model is the most effective among all methods. Furthermore, it was performed on the state-of-the-art TuSimple dataset, as shown in Table 3.

**Table 3:** Comparison between our model and other methods based on TuSimple dataset.

Model	Accuracy (%)	FP	FN
ResNet-18^4^	93.78	0.1035	0.0964
ResNet-34^4^	94.66	0.0804	0.0775
LaneNet^2^	93.38	0.0780	0.0224
SCNN^1^	95.12	0.0610	0.0643
PolyLaneNet^3^	93.36	0.0942	0.0933
ERFNet^6^	94.34	0.0850	0.0777
ENet^5^	94.68	0.0977	0.0603
ESCN model based on Resnet-34(ours)	95.49	0.0307	0.0342
Full Matrix Channel and Spatial attention based on ResNet-34(ours)	95.30	0.0322	0.0368

2. Results on CULane dataset.

With the CULane benchmark, we also know that the accuracy of our proposed model outperforms all other methods. In nine challenging scenarios, it outperforms all other methods, namely 75.67%. We can see that it increases by 13.48% in the total scenario compared to ResNet-18^4^ and increases by 4.17% for R-34-E2E, which is the highest among all methods. In the cross scenario, the values are FP, the value of our model is also the lowest. We know for embedded system run time is priority. Although the inference speed of our model is slower than ResNet-18, it significantly outperforms its accuracy. For embedded systems, real-time performance is a top priority. According to the comparison of FPS indicators in Table 4, the speed of our recommended model is better than most models. In the FPS comparison, our proposed model is 82.84 faster than the slowest model and only 33.12 slower than the fastest model. However, the accuracy of our model is 13.14% higher than that of the fastest model.

**Table 4:** Accuracy comparison between our model and other methods based on CULane dataset.

Model	Total (%)	Normal (%)	Crowd (%)	Highlight (%)	Shadow (%)	Arrow (%)	Curve (%)	Cross	Night (%)	No line (%)	FPS
SCNN^1^	68.39	87.59	66.64	57.53	57.95	81.88	63.14	2079	62.26	39.76	7.5
ResNet-18^4^	62.19	82.69	59.11	48.71	53.13	73.36	57.47	1922	56.49	31.63	**123.46**
ResNet-34^4^	66.23	86.74	63.27	56.60	62.83	78.02	59.39	2571	59.96	37.93	67.74
Res18-VP^56^	69.1	89.2	61.9	59.3	**81.6**	59.3	60.8	2919	62.6	41.7	75.54
ENet^5^	63.83	83.99	62.60	51.18	55.13	73.81	59.65	3657	57.81	36.52	43.68
Res18-ultra^57^	68.4	87.7	66.0	58.4	62.80	81.0	57.9	1743	62.1	40.2	23.46
ERFNet^6^	69.28	88.97	67.09	58.10	60.38	80.53	62.86	3363	64.88	43.05	85.87
FastDraw^58^	–	85.9	63.6	57.0	59.9	79.4	65.2	7013	57.8	40.6	63.26
R-34-SAD^59^	70.70	89.90	68.50	59.90	67.70	83.80	66.02	1960	64.60	42.20	75
R-34-E2E^6^	71.50	90.40	69.90	61.50	68.10	83.70	**69.80**	2077	63.20	45.01	57.43
ESCN model based on Resnet-34(ours)	75.32	91.23	73.03	**66.04**	71.56	**86.63**	66.92	1175	69.46	**48.10**	90.34
Full Matrix Channel and Spatial attention based on ResNet-34(ours)	**75.67**	**91.34**	**73.21**	66.02	72.01	86.45	67.01	**1157**	**69.57**	47.98	65.36

Significant values are in [bold].

**Figure 5:**
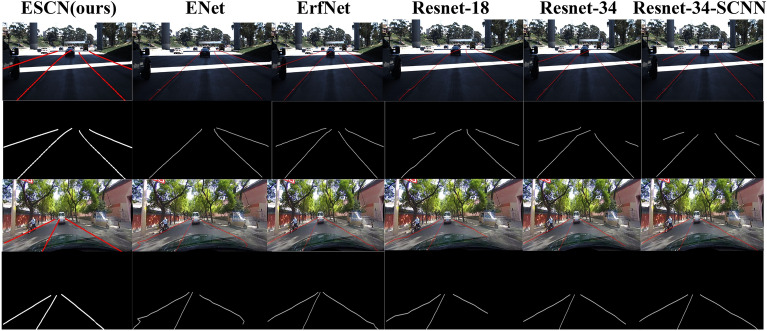
Comparison between our proposed model and other methods in visualization based on CULane benchmark.

From Fig. 5, we can easily see that the lane marking line detection results of our proposed model are better than those of the other visualization methods. For example, from the visualization results, it is not difficult to see that other models predict lane lines. Most lane lines have jitter and position deviation, and the lane lines cannot have a parallel relationship. Therefore, it is obvious that our proposed model also achieves the state-of-the-art performance on the CULane benchmark as shown in Table 4.

### Ablation study

Conv1d and Conv2d were used instead of the full matrix in our proposed model. Our goal is to reduce the number of calculations and parameters to see if we can achieve the effect of not significantly reducing the accuracy of the model. Judging from the actual comparison results in Table 5, when the channel-related self-attention mechanism is optimized, the F1 index is only reduced by 0.35, which is about 0.46%. Table 5 shows that our ESCN model performs better than the entire matrix model in the TuSimple test. Accuracy improved by 0.19%. FP and FN received promotions. The values increased by 0.0015 and 0.0026 respectively. In the CULane benchmark, we can also see that although F1 decreased by 0.45%, as shown in Tables 5 and 6, convolution was used instead of matrix calculation, which significantly reduced the calculation.

**Table 5:** Ablation comparison on TuSimple benchmark dataset.

Model	Accuracy (%)	FP	FN
ESCN model based on Resnet-34(ours)	**95.49**	**0.0307**	**0.0342**
Full Matrix Channel and Spatial attention based on ResNet-34(ours)	95.30	0.0322	0.0368

Significant values are in [bold].

**Table 6:** Ablation comparison on CULane benchmark dataset.

Model	TP	FP	FN	Precision (%)	Recall (%)	F1 (%)
ESCN model based on Resnet-34(ours)	**72,383**	14,910	32,503	82.91	69.01	75.32
Full Matrix Channel and Spatial attention based on ResNet-34(ours)	72,611	**14,401**	**32,275**	**83.44**	**69.22**	**75.67**

Significant values are in [bold].

**Figure 6:**
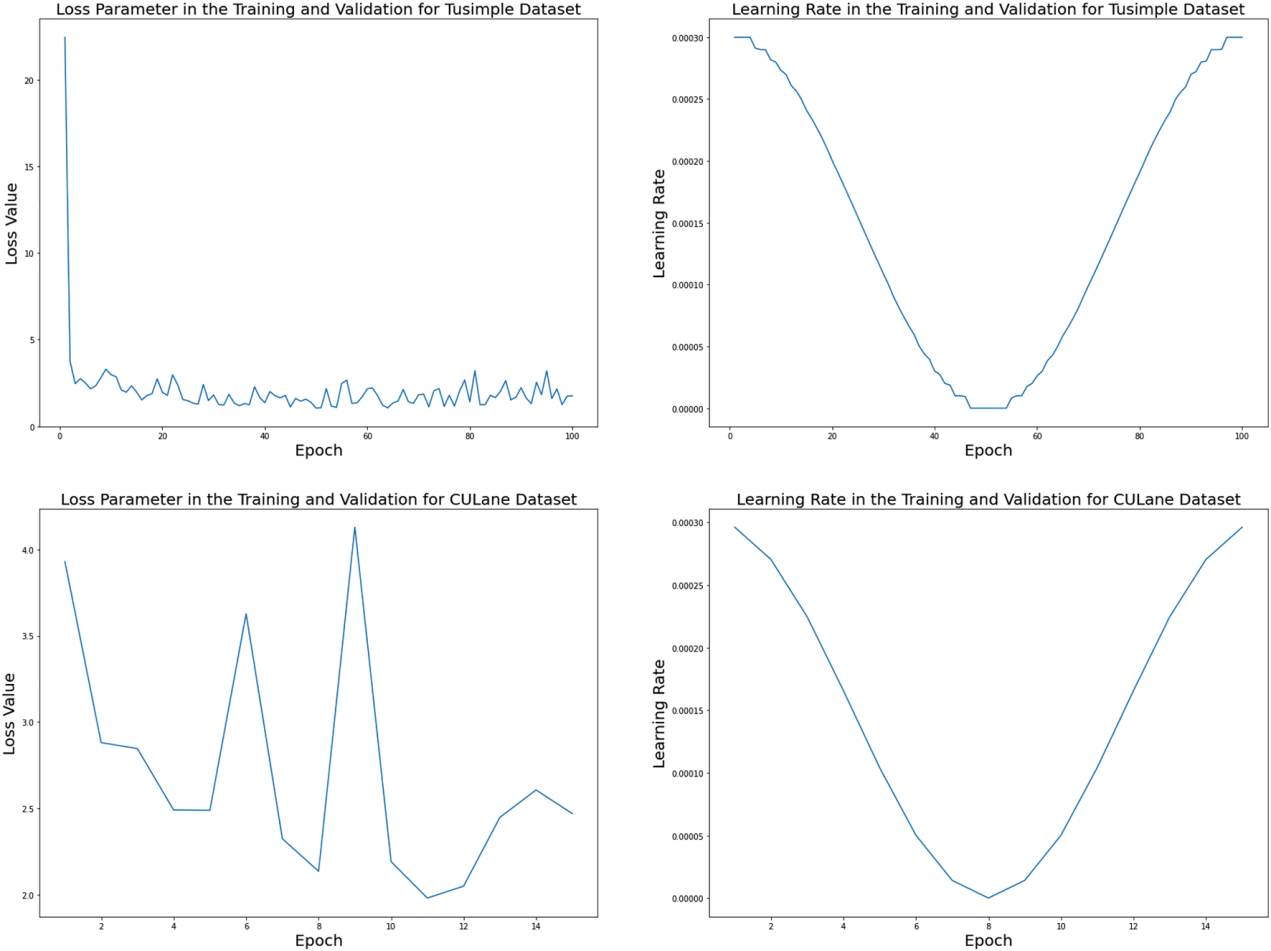
The curve of loss parameter and learning rate parameter in TuSimple dataset and CULane dataset.

From Fig. 6, we can easily see that the loss parameter changes rapidly at the beginning of the training phase, regardless of whether it is the Tusimple dataset or the CULane dataset. The loss change gradually stabilizes for the Tusimple dataset. However, the loss changes of the CULane dataset are still quite intense. For the learning rate parameter, whether it is the Tusimple data set or the CULane data set, their changes are basically the same, they gradually become smaller and then gradually increase.

## Conclusion

In this study, we propose an effective spatial and channel network aimed at detecting lane marking lines, especially in difficult environments. We used channel self-attention, which deals with global and contextual information in a single channel, and spatial self-attention, which focuses more on location in many channels, to identify aspects that are likely to be missed in difficult situations. We evaluated our proposed model using the TuSimple and CULane benchmarks. With an individual advantage of 3.82% and 0.37% over all other methods. Undoubtedly, it delivers excellent performance. However, there are also three limitations in our proposed model. First, we only simplify the channel-wise attention model through a global average pooling operation. Although it promotes inference speed, it reduces accuracy compared to the fully connected matrix. Second, we take the same measures for the spatial channel. As a result, global information is easily ignored and the relationship between different pixels is missing. In addition, the kernel size limits the receptive field. Finally, our proposed model pays significantly more attention to the information in an image while neglecting the associated connection between continuous images. We now also understand that the self-attention mechanism is only able to retrieve global information across channels and locations, but not semantic and contextual information between frames. Accuracy in the lane marking detection phase is crucial for autonomous driving. Runtime applications on embedded platforms are a crucial part of future research. In future work, we will consider a comprehensive technology to construct our model. Like rnn, lstm, semantic segmentation and instance segmentation, we will combine them with attention or self-attention to combine their advantages and obtain an improved model. If we don’t limit ourselves to compatibility for embedded systems, we also consider large models.

## Data Availability

The data presented in this study are openly available in https://github.com/TuSimple/tusimple-benchmark and https://xingangpan.github.io/projects/CULane.html.

## References

[1] Pan, X., Shi, J., Luo, P., Wang, X. & Tang, X.: Spatial as deep: Spatial cnn for traffic scene understanding. In *Proceedings of the AAAI Conference on Artificial Intelligence* 7276–7283 (2018).

[2] Tabelini, L., Berriel, R., Paixao, T. M. *et al*. Polylanenet: Lane estimation via deep polynomial regression. In *25th International Conference on Pattern Recognition (ICPR)* 6150–6156 (IEEE, 2020).

[3] Wang, Z., Ren, W. & Qiu, Q. Lanenet: Real-time lane detection networks for autonomous driving. arXiv 180701726 (2018).

[4] He, K., Zhang, X., Ren, S. & Sun, J. Deep residual learning for image recognition. In *CVPR* (2016).

[5] Paszke, A., Chaurasia, A., Kim, S. & Culurciello, E. ENet: A deep neural network architecture for real-time semantic segmentation. arXiv:1606.02147, (2016).

[6] Liu, T., Chen, Z., Yang, Y., Wu, Z. & Li, H. Lane detection in low-light conditions using an efficient data enhancement: Light conditions style transfer. arXiv:2002.01177, (2020).

[7] Narote SP, Bhujbal PN, Narote AS, Dhane DM (2018). A review of recent advances in lane detection and departure warning system. Pattern Recogn..

[8] Niu J, Lu J, Xu M, Lv P, Zhao X (2016). Robust lane detection using two-stage feature extraction with curve fitting. Pattern Recogn..

[9] Dewangan, D. K. & Sahu, S. P. Lane detection for intelligent vehicle system using image processing techniques. In *Data Science. Transactions on Computer Systems and Networks* (eds. Verma, G. K. e*t al*.) (Springer, 2021). 10.1007/978-981-16-1681-5_21.

[10] Selver MA, Er E, Belenlioglu B, Soyaslan Y (2016). Camera based driver support system for rail extraction using 2-D gabor wavelet decompositions and morphological analysis. Proc. IEEE Conf. Intell. Rail Transp..

[11] Lowe, D. G. Object recognition from local scale-invariant features. In *Proceeding of the IEEE International Conference on Computer Vision* (1999).

[12] Bay, H., Tuytelaars, K. & Van Gool, L. Surf: Speeded up robust features. In *Proceedings of the European Conference on Computer Vision* (2006).

[13] Chen, Y., Kalantidis, Y., Li, J., Yan, S. & Feng, J. A2-Nets: Double attention networks. In *NIPS* (2018).

[14] Hu, J., Shen, L. & Sun, G. Squeeze-and-excitation networks. In *CVPR* (2018). 10.1109/TPAMI.2019.2913372 31034408

[15] Woo, S., Park, J., Lee, J.-Y. & Kweon, I. S. CBAM: Convolutional block attention module. In *ECCV* (2018).

[16] Hu, J., Shen, L., Albanie, S., Sun, G. & Vedaldi, A. Gather-excite: Exploiting feature context in convolutional neural networks. In *NeurIPS* (2018).

[17] Fu, J. *et al*. Dual attention network for scene segmentation. In *CVPR* (2019).

[18] Gao, Z., Xie, J., Wang, Q. & Li, P. Global second-order pooling convolutional networks. In *CVPR* (2019).

[19] Aly, M. Real time detection of lane markers in urban streets. In *Proc. IEEE Intell. Vehicles Symp.* 7–12 (2008).

[20] McCall J, Trivedi M (2006). Video-based lane estimation and tracking for driver assistant: Survey, system, and evaluation. IEEE Trans. Intell. Transp. Syst..

[21] Zhou, S. *et al*. A novel lane detection based on geometrical model and gabor filter. In *IEEE Intelligent Vehicles Symposium* 59–64 (2010).

[22] Sun, T.-Y., Member IEEE, Tsai, S.-J. & Chan, V. HSI color model based lane-marking detection. In *2006 IEEE Intelligent Transportation Systems Conference* 1168–1173 (2006).

[23] Yu, B. & Jain, A. K. Lane boundary detection using a multi resolution Hough transform. In *Proceedings of International Conference on Image Processing* (2022). 10.1109/ICIP.1997.638604,2022.08.

[24] Wang Y, Teoh EK, Shen D (2008). Robust lane detection and tracking in challenging scenarios. IEEE Trans. Intell. Transp. Syst..

[25] Alvarez, J. M. & Lopez, A. Novel index for objective evaluation of road detection algorithms. In *Proc. of the IEEE Intelligent Transportation Systems* (2008).

[26] Wang, Y., Shen, D. & Teoh, E. K. Lane detection using Catmull-Rom spline. In *Proc. of the IEEE Intelligent Vehicles* (1998).

[27] Zhou, S. *et al*. A novel lane detection based on geometrical model and gabor filter. In *2010 IEEE Intelligent Vehicles Symposium University of California, San Diego, CA, USA* (2010).

[28] Marr D, Hildreth E (1980). “Theory of edge detection. Proc. R. Soc. Lond. Ser. B Biol. Sci..

[29] Liu, W., Zhang, H., Duan, B., Yuan, H. & Zhao, H. Vision-based real-time lane marking detection and tracking. In *Proc. of the IEEE Intelligent Transportation Systems* (2008).

[30] Goldbecka J, Huertgena B, Ernsta S, Kelchb L (2000). Lane following combining vision and DGPS. Image Vis. Comput..

[31] Seo, D. & Jo, K. Inverse perspective mapping based road curvature estimation. In *System Integration (SII), 2014 IEEE/SICE International Symposium on, Dec 2014* 480–483 (2014).

[32] Berriel, R. F., Aguiar, E., Filho, V. V. S. & Oliveira-Santos, T. A particle filter-based lane marker tracking approach using a cubic spline model. In *2015 28th SIBGRAPI Conference on Graphics, Patterns and Images, Date of Conference: 26–29 August 2015* (2015). 10.1109/SIBGRAPI.2015.15.

[33] Shi, J. & Tomasi, C. Good features to track. In *Proc. of the IEEE Conference on Computer Vision and Pattern Recognition* 593–600 (1994).

[34] Lookingbill, A., Lieb, D. & Thrun, S. Optical flow approaches for [34]self-supervised learning in autonomous mobile robot navigation. In *Autonomous Navigation in Dynamic Environments* (2007).

[35] Bouguet, J. *Pyramidal Implementation of the Lucas Kanade Feature Tracker Description of the Algorithm*. (Intel Corporation, Microprocessor Research Labs, 2000).

[36] Szegedy, C. *et al*. *Scalable, High-Quality Object Detection*. arXiv:1412.1441 (2014).

[37] Dewangan DK, Sahu SP, Sairam B (2021). VLDNet: Vision-based lane region detection network for intelligent vehicle system using semantic segmentation. Computing.

[38] Kim J, Lee M (2014). Robust lane detection based on convolutional neural network and random sample consensus. ICONIP Neural Inf. Process..

[39] Huval, B. *et al*. An empirical evaluation of deep learning on highway driving. arXiv:1504.01716 (2015).

[40] Girshick, R. *et al*. Rich feature hierarchies for accurate object detection and semantic segmentation. In *Computer Vision and Pattern Recognition (CVPR), 2014 IEEE Conference on IEEE* (2014).

[41] Dewangan DK, Sahu SP (2023). Lane detection in intelligent vehicle system using optimal 2-tier deep convolutional neural network. Multimed. Tools Appl..

[42] Badrinarayanan, V., Kendall, A. & Cipolla, R. Segnet: A deep convolutional encoder-decoder architecture for scene segmentation. In *IEEE Transactions on Pattern Analysis and Machine Intelligence, vol. 99* 1–1 (2017). 10.1109/TPAMI.2016.2644615 28060704

[43] Ronneberger, O., Fischer, P. & Brox, T. *U-Net: Convolutional Networks for Biomedical Image Segmentation* (Springer, 2015).

[44] Kim J, Park C (2017). End-to-end ego lane estimation based on sequential transfer learning for self-driving cars. IEEE Conf. Comput. Vis. Pattern Recogn. Workshops (CVPRW).

[45] Bruls T, Maddern W, Morye AA, Newman P (2018). Mark yourself: Road marking segmentation via weakly-supervised annotations from multimodal data. IEEE Int. Conf. Robot. Autom. (ICRA).

[46] Neven, D., De Brabandere, B., Georgoulis, S., Proesmans, M. & Van Gool, L. Towards end-to-end lane detection: An instance segmentation approach. In *IEEE Intell. Veh. Symp.* 286–291 (2018).

[47] Qin, Z., Wang, H. & Li, X. *Ultra Fast Structure-aware Deep Lane Detection*. arXiv:2004.11757v4 (2020).

[48] Hou Y, Ma Z, Liu C, Loy CC (2019). Learning lightweight lane detection CNNs by self attention distillation. Int. Conf. Computer. Vis..

[49] Tabelini L, Berriel R, Pao TM, Badue C, Souza AFD, Oliveira-Santos T (2021). Keep your eyes on the lane: Real-time attention-guided lane detection. IEEE Conf. Comput. Vis. Pattern Recog..

[50] Yao Z, Chen X (2022). Efficient lane detection technique based on lightweight attention deep neural network. J. Adv. Transp..

[51] Wang B, Yan X, Li D (2022). An end-to-end lane detection model with attention and residual block. Comput. Intell. Neurosci..

[52] Lee, M. *et al*. Robust lane detection via expanded self attention. In *IEEE/CVF Winter Conference on Applications of Computer Vision (WACV)* (2022). 10.48550/arXiv.2102.07037.

[53] Zhao Q, Peng Q, Zhuang Y (2022). Lane line detection based on the codec structure of the attention mechanism. J. Real-Time Image Process..

[54] Lin, T.-Y., Goyal, P., Girsshcik, R., He, K. & Dollar, P. Focal loss for dense object detection. In *Conference Computer Vision and Pattern Recognition (CVPR)* (2017). 10.1109/TPAMI.2018.2858826 30040631

[55] TuSimple. Tusimple Benchmark (2022, accessed 12 Oct 2022). https://github.com/TuSimple/tusimple-benchmark.

[56] Liu, Y.-B., Zeng, M. & Meng, Q.-h. Heatmap-based vanishing point boosts lane detection. arXiv (2020).

[57] Qin, Z., Wang, H. & Li, X. Ultra fast structure-aware deep lane detection. In *Computer Vision–ECCV 2020: 16th European Conference, Glasgow, Proceedings, Part XXIV* 16 (Springer International Publishing, 2020).

[58] Philion, J. Fastdraw: Addressing the long tail of lane detection by adapting a sequential prediction network. In *Proceedings of the IEEE/CVF Conference on Computer Vision and Pattern RecognitionLong Beach, CA, USA* (2019).

[59] Wang Y, Shen D, Teoh EK (2000). Lane detection using spline model. Pattern Recogn. Lett..

